# Acute Kidney Injury-Induced Circulating TNFR1/2 Elevations Correlate with Persistent Kidney Injury and Progression to Fibrosis

**DOI:** 10.3390/cells12182214

**Published:** 2023-09-05

**Authors:** Akshayakeerthi Arthanarisami, Yohei Komaru, Charikleia Katsouridi, Julian Schumacher, Deborah K. Verges, Liang Ning, Mai M. Abdelmageed, Andreas Herrlich, Eirini Kefaloyianni

**Affiliations:** 1 Department of Medicine, Washington University in St. Louis, St. Louis, MO 63110, USA; akshayakeerthi1997@gmail.com (A.A.); ykomaru@wustl.edu (Y.K.); charikleia@wustl.edu (C.K.); juluan.schumacher@gmail.com (J.S.); debbieverges@att.net (D.K.V.); liang.ning@wustl.edu (L.N.); mai.maguid@gmail.com (M.M.A.); aherrlich@wustl.edu (A.H.); 2 VA St. Louis Health Care System, John Cochran Division, St. Louis, MO 63106, USA

**Keywords:** circulating TNFR1/2, kidney injury, progression, fibrosis

## Abstract

Elevated levels of circulating tumor necrosis factor receptors 1 and 2 (cTNFR1/2) predict chronic kidney disease (CKD) progression; however, the mechanisms of their release remain unknown. Whether acute kidney injury (AKI) drives cTNFR1/2 elevations and whether they predict disease outcomes after AKI remain unknown. In this study, we used AKI patient serum and urine samples, mouse models of kidney injury (ischemic, obstructive, and toxic), and progression to fibrosis, nephrectomy, and related single-cell RNA-sequencing datasets to experimentally test the role of kidney injury on cTNFR1/2 levels. We show that TNFR1/2 serum and urine levels are highly elevated in all of the mouse models of kidney injury tested, beginning within one hour post injury, and correlate with its severity. Consistent with this, serum and urine TNFR1/2 levels are increased in AKI patients and correlate with the severity of kidney failure. Kidney tissue expression of TNFR1/2 after AKI is only slightly increased and bilateral nephrectomies lead to strong cTNFR1/2 elevations, suggesting the release of these receptors by extrarenal sources. The injection of the uremic toxin indoxyl sulfate in healthy mice induces moderate cTNFR1/2 elevations. Moreover, TNF neutralization does not affect early cTNFR1/2 elevations after AKI. These data suggest that cTNFR1/2 levels in AKI do not reflect injury-induced TNF activity, but rather a rapid response to loss of kidney function and uremia. In contrast to traditional disease biomarkers, such as serum creatinine or BUN, cTNFR1/2 levels remain elevated for weeks after severe kidney injury. At these later timepoints, cTNFR1/2 levels positively correlate with remaining kidney injury. During the AKI-to-CKD transition, elevations of TNFR1/2 kidney expression and of cTNFR2 levels correlate with kidney fibrosis levels. In conclusion, our data demonstrate that kidney injury drives acute increases in cTNFR1/2 serum levels, which negatively correlate with kidney function. Sustained TNFR1/2 elevations after kidney injury during AKI-to-CKD transition reflect persistent tissue injury and progression to kidney fibrosis.

## 1. Introduction

Since initial reports that circulating tumor necrosis factor receptors 1 and 2 (cTNFR1/2) elevations predict diabetic nephropathy progression [1,2], numerous studies have demonstrated that cTNFR1/2 level increases correlate with the severity and progression of CKD of any cause examined (e.g., in additional diabetic nephropathy populations, idiopathic membranous nephropathy, IgA nephropathy, lupus nephritis, and hypertension-related nephropathy [3,4,5,6,7,8,9,10,11,12]). Elevated cTNFR1/2 levels also correlate with mortality in end-stage renal disease patients, and with nephropathy, cardiovascular events and mortality in diabetes [13,14,15]. Despite their clinical importance as markers of kidney disease progression, very little is known about the mechanisms of their release.

AKI episodes can lead to CKD development and/or enhance CKD progression. Recent studies showed increases of cTNFR1/2 levels in patients who had an AKI episode while hospitalized for CKD [16], critical illness [17,18], cardiovascular disease [19], and/or other etiologies [20,21], and significant correlations with disease outcomes. It is still, however, not known whether kidney injury alone can induce TNFR1/2 elevations and whether their levels change in the AKI-to-CKD transition. Moreover, we do not have any evidence of the cause of cTNFR1/2 elevations in kidney disease. For example, it has been speculated that cTNFR1/2 represent markers of TNF pathway activation in kidney or other diseases [22,23], but this has not been experimentally tested. Although there are numerous studies that examine the kidney mRNA and protein levels of TNFR1/2 in animal models of kidney disease, to our knowledge their circulating levels have not yet been tested in any of these animal models and all information on their elevations to date come from human biomarker studies.

In this study, we used different mouse models of kidney disease to experimentally test the role of kidney injury on TNFR1/2 levels. We also used AKI patient samples to confirm our findings in human disease. We further examined the potential contribution of TNF and uremia on cTNFR1/2 elevations and the relationship of cTNFR1/2 with sustained kidney injury after AKI and with progression to fibrosis (AKI-to-CKD).

## 2. Materials and Methods

### 2.1. Human Samples

Deidentified human samples of patients with AKI without CKD, multiorgan failure, or healthy controls were provided by the Kidney Translational Research Center (KTRC), under a protocol approved by the Washington University Institutional Review Board (IRB 201102312). Informed consent was obtained for the use of data and samples for all participants at Washington University. The cause of AKI was as follows: 15 acute tubular necrosis (ATN), one oxalate crystal ATN, one AKI of obstructive etiology, one contrast nephropathy, one ureterovesical junction stone AKI, one AKI with HIV, one AKI with malignant hypertension, one acute interstitial nephritis, one AKI with atypical hemolytic uremic syndrome, one AKI with hepatitis C-associated glomerulonephritis. AKI patients did not have clinical evidence of other organ failure based on clinical data review and serum biochemistries.

### 2.2. Animal Experiments

8–12-week-old male mice from Jackson Laboratories were used in accordance with the animal care and use protocol approved by the Institutional Animal Care and Use Committee of the Washington University School of Medicine, in adherence to standards set in the Guide for the Care and Use of Laboratory Animals. Strains were: C57Bl/6J (used in all experiments in this study, unless noted) and FVB/N (used to confirm results in one experiment). Kidney ischemia at 37 °C for 18–27 min, as described in the text, was induced bilaterally using the flank approach [24]. A group of mice was subjected to unilateral ischemia (left kidney) for 25 min. UUO was executed as described previously [25,26]. Sham-operated mice underwent the same surgical procedure except for the ischemia/ureter ligation.

Acute aristolochic acid nephropathy was induced in C57Bl/6J mice by a one-time IP injection of aristolochic acid (5, 10, or 20 mg per kg body weight, as described in the text, Millipore-Sigma, Burlington, MA, USA) in PBS. Cisplatin nephropathy was induced in C57Bl/6J mice by a one-time IP injection of cisplatin (20 mg per kg body weight, Millipore-Sigma) in PBS. Indoxyl sulfate was injected once at a concentration of 100 or 200 mg/kg in PBS IP, as described previously [27,28]. Murine etanercept (Pfizer, New York City, NY, USA) 12.5 mg/mL in PBS was injected into FVB/N mice at 10 mg per kg IP 2 h before surgery [29]. Anti-TNF antibody (MP6-XT22, eBioscience-ThermoFisher, Waltham, MA, USA) was diluted in PBS and injected into animals at 10 mg per kg IP 2 h before surgery. Control mice were administered the same amount of vehicle or control IgG, in PBS.

Each mouse sample was assigned a unique numeric identifier that did not contain information on the sample identity (mouse genotype, surgery type/treatment, etc.) to eliminate bias during downstream analysis.

### 2.3. Renal Function

Serum creatinine was measured by LC–mass spectrometry at the O’Brien Core Center for AKI Research (University of Alabama School of Medicine, Birmingham, AL, USA). BUN levels were measured by DIUR-100 kit (BioAssay Systems, Hayward, CA, USA).

### 2.4. ELISA

Circulating TNFR1 and TNFR2 were measured in human or mouse samples using enzyme-linked immunosorbent assay (ELISA) kits (DY255, DY726, DY425, and DY426, respectively, all from R&D Systems, Minneapolis, MN, USA). Serum dilutions for ELISAs were 100-fold for mouse and 50-fold for human samples. Urinary measurements were corrected for urinary creatinine, measured by the DICT-500 kit (BioAssay Systems).

### 2.5. Single-Cell RNA Sequencing (scRNAseq)

Mouse: scRNAseq analysis was conducted on kidney samples from sham and AKI as previously described [30]. The expression levels of *Tnfrsf1a* (TNFR1) and *Tnfrsf1b* (TNFR2) in each cluster were visualized using ‘plot1cell’ package [31].

Human: scRNAseq dataset from human kidney biopsy samples was obtained from the Kidney Precision Medicine Project (KPMP) website in Seurat object format (https://www.kpmp.org/; accessed on 24 March 2022). This publicly available dataset included 20 participants in the healthy reference group and 12 in the AKI group. We performed cell-type annotation following the original labeling in the project while excluding degenerative cells with marked loss of differentiation markers and/or increased %endoplasmic reticulum (ER) or %mitochondrial genes. Clusters with less than 10 cells in either the healthy or AKI group were removed. The expression levels of *Tnfrsf1a* (TNFR1) and *Tnfrsf1b* (TNFR2) were visualized by cluster [31].

### 2.6. qPCR

Total RNA was isolated from mouse kidneys (whole kidney or cortex) using Trizol (Invitrogen, Waltham, MA, USA) following the manufacturers’ instructions. Total RNA was reverse transcribed using the QuantiTect RT Kit (QIAGEN, Germantown, MD, USA) and real-time polymerase chain reaction (PCR) was performed with Fast SYBR Green (QIAGEN). *Gapdh* was used as the housekeeping gene. Primer sequences are provided in Supplementary Table S1. Data were analyzed using the ΔΔCt method.

### 2.7. Immunofluorescence Staining

Immunofluorescence staining of the kidney was performed on frozen sections following standard protocols, as previously described [26]. For each kidney staining, ten images per kidney throughout the tissue were collected for blinded quantification. Images were analyzed using the Fiji software (ImageJ2 v2.14.0).

### 2.8. Histology

Kidney histology was examined in formalin-fixed sections. Fibrosis severity was quantified in the kidney cortex by measuring the Sirius red–stained area using the Fiji software. Ten images per kidney throughout the tissue were collected for blinded quantification.

### 2.9. Statistics

All results are reported as the mean ± SEM. Comparison of 2 groups was performed using an unpaired, 2-tailed *t*-test or a Pearson correlation analysis where appropriate. Comparison of 3 or more groups was performed via ANOVA and Tukey’s post hoc test. Statistical analyses were performed using GraphPad Prism 9.0 (GraphPad Software Inc. Boston, MA, USA). A *p* value of less than 0.05 was considered significant.

## 3. Results

### 3.1. Circulating TNFR1/2 Levels Are Increased in Mouse Models of AKI and Correlate with Kidney Injury Levels

**Figure 1:**
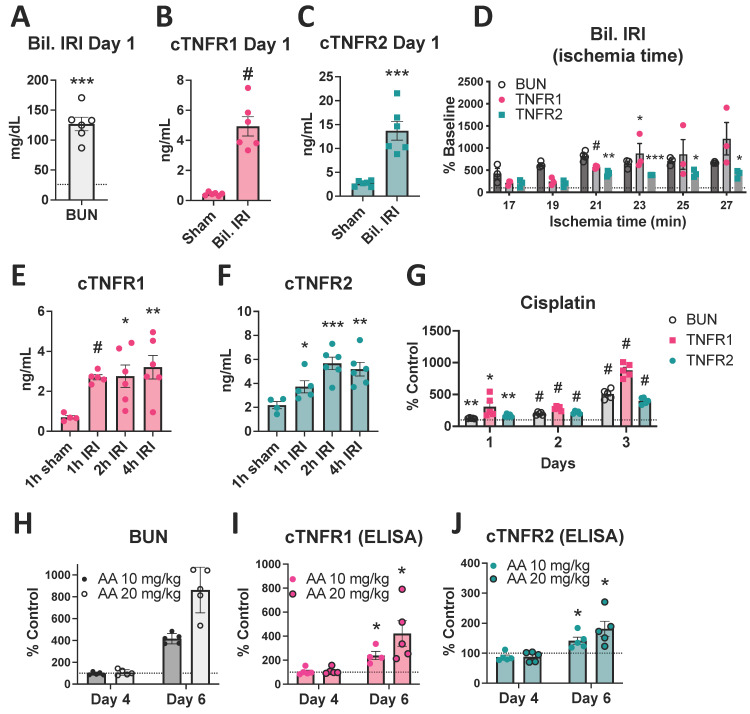
Circulating TNFR1/2 are increased in mouse AKI models, and their levels correlate with kidney injury levels. (**A**) BUN levels at day 1 post injury in the severe bilateral IRI model (the dotted line denotes baseline levels). (**B**) cTNFR1 levels at day 1 post bilateral IRI or sham surgery. (**C**) TNFR2 levels at day 1 post bilateral IRI or sham surgery. (**D**) BUN, cTNFR1, and cTNFR2 levels at day 1 post bilateral IRI of increasing ischemia duration, expressed as percent over their respective baseline levels. (**E**) cTNFR1 levels measured at different time points (1–4 h) post severe bilateral IRI or sham. (**F**) cTNFR2 levels measured at different time points (1–4 h) post severe bilateral IRI or sham. (**G**) BUN, cTNFR1, and cTNFR2 levels measured at different time points (1–3 days) after cisplatin injection expressed as percent over vehicle (% control) levels. (**H**) BUN, (**I**) cTNFR1, and (**J**) cTNFR2 levels measured at different time points (4 or 6 days) after 10 or 20 mg/kg aristolochic acid (AA) administration, expressed as percent over their respective baseline levels. *: *p* < 0.05, **: *p* < 0.01, ***: *p* < 0.001, #: *p* < 0.0001.

To test whether kidney injury can drive cTNFR1/2 elevations, we first measured cTNFR1/2 levels in the ischemia-reperfusion injury (IRI) mouse model. This model was chosen because the level and timing of injury can be precisely controlled. We found that severe bilateral IRI, confirmed by BUN elevations above 100 mg/dL at 24 h post injury (Figure 1A), results in significant elevations of cTNFR1 and cTNFR2 levels 24 h post IRI, approximately by 10- and 5-fold over their respective baseline levels (Figure 1B,C). Increasing ischemic injury, induced by increasing ischemia time and confirmed by increasing BUN elevations, results in increasing cTNFR1/2 concentrations, up to a plateau level (Figure 1D). At earlier time points post injury, cTNFR1/2 levels show a gradual increase, starting already at 1 h post IRI (Figure 1E,F). To test whether cTNFR1/2 elevations represent a universal AKI outcome and not a specific response to ischemia/reperfusion, we used the nephrotoxic models of cisplatin and aristolochic acid. We found that both treatments result in increased cTNFR1/2 levels that mirror the course of kidney injury as assessed by BUN elevations (Figure 1G–J). A moderate (10 mg/kg) and high (20 mg/kg) dose of aristolochic acid resulted in moderate and severe injury, respectively (assessed by BUN), and in corresponding cTNFR1/2 elevations (Figure 1H–J), confirming that increased severity of kidney injury results in increased cTNFR1/2 elevations.

**Figure 2:**
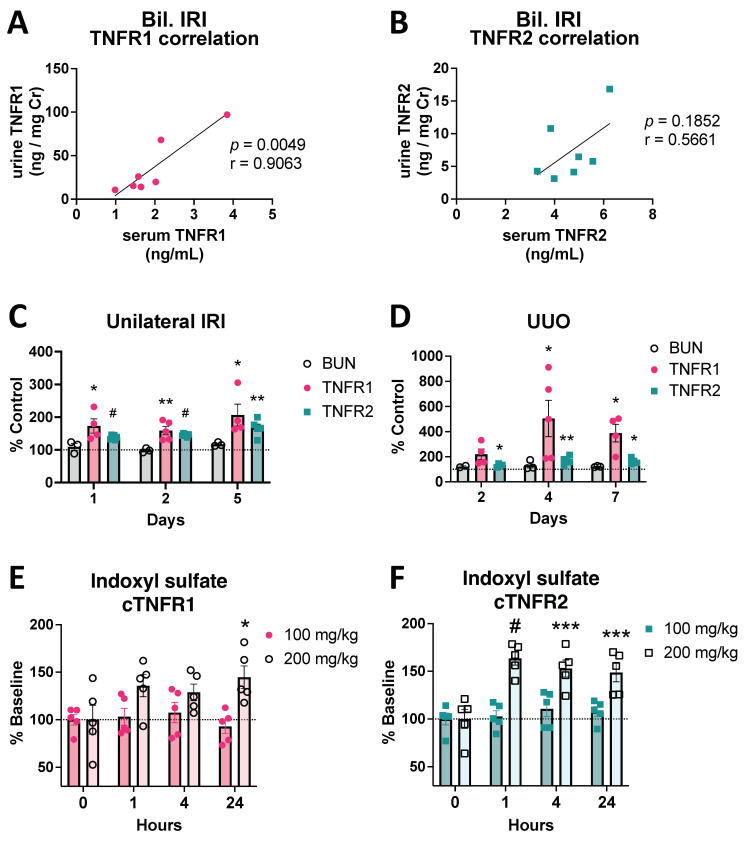
Urinary TNFR1/2 levels are increased by IRI, correlating to their serum levels, and cTNFR1/2 are increased unilaterally in kidney injury models. (**A**) Correlation of urinary TNFR1 levels with serum TNFR1 levels in matching samples, at day 1 post severe bilateral IRI. (**B**) Correlation of urinary TNFR2 levels with serum TNFR2 levels in matching samples, at day 1 post severe bilateral IRI. (**C**) BUN, cTNFR1, and cTNFR2 levels measured at different time points (1–5 days) after unilateral IRI. (**D**) BUN, cTNFR1, and cTNFR2 levels measured at different time points (2–7 days) after unilateral ureteral obstruction (UUO). (**E**) cTNFR1 and (**F**) cTNFR2 levels after 100 or 200 mg/kg indoxyl sulphate administration, expressed as percent over their respective baseline levels. Pearson correlation (r) value and *p* values are shown in (**A**,**B**). *: *p* < 0.05, **: *p* < 0.01, ***: *p* < 0.001, #: *p* < 0.0001.

To determine whether the observed cTNFR1/2 increases were a result of reduced receptor clearance or increased production/release, we first tested TNFR1/2 urine levels in our AKI models. We found significant urinary TNFR1/2 elevations 24 h post bilateral IRI that correlate with cTNFR1/2 serum levels (Figure 2A,B), indicating that their overall levels (serum and urine) increase after injury. We also tested cTNFR1/2 serum levels in unilateral kidney injury models, in which function in the uninjured kidney compensates for loss of function in the injured kidney, and thus the overall kidney clearance of the receptors should be fairly intact. Both unilateral IRI and unilateral ureteral obstruction (UUO) result in significant elevations of cTNFR1/2, although BUN levels remain at baseline in both models (Figure 2C,D). We further hypothesized that cTNFR1/2 levels could be directly affected by uremic toxins, which are elevated during kidney function loss. To test this hypothesis, we exposed healthy mice to increasing doses of indoxyl sulfate (IS), a protein-bound uremic toxin that is markedly elevated in the plasma of kidney disease patients and can affect the function of many cell types, including tubular, immune, and endothelial cells [32,33]. We found that a single IS injection caused moderate dose-dependent elevations of both cTNFR1/2, with cTNFR2 showing significant elevations within one hour after injection (Figure 2E,F). Taken together, these results suggest that cTNFR1/2 levels increase, at least in part, secondary to increased production/release. However, decreased receptor clearance by the injured kidney may also contribute to their observed elevations.

### 3.2. Human AKI Leads to Increased cTNFR1/2 Levels That Correlate with AKI Severity

**Figure 3:**
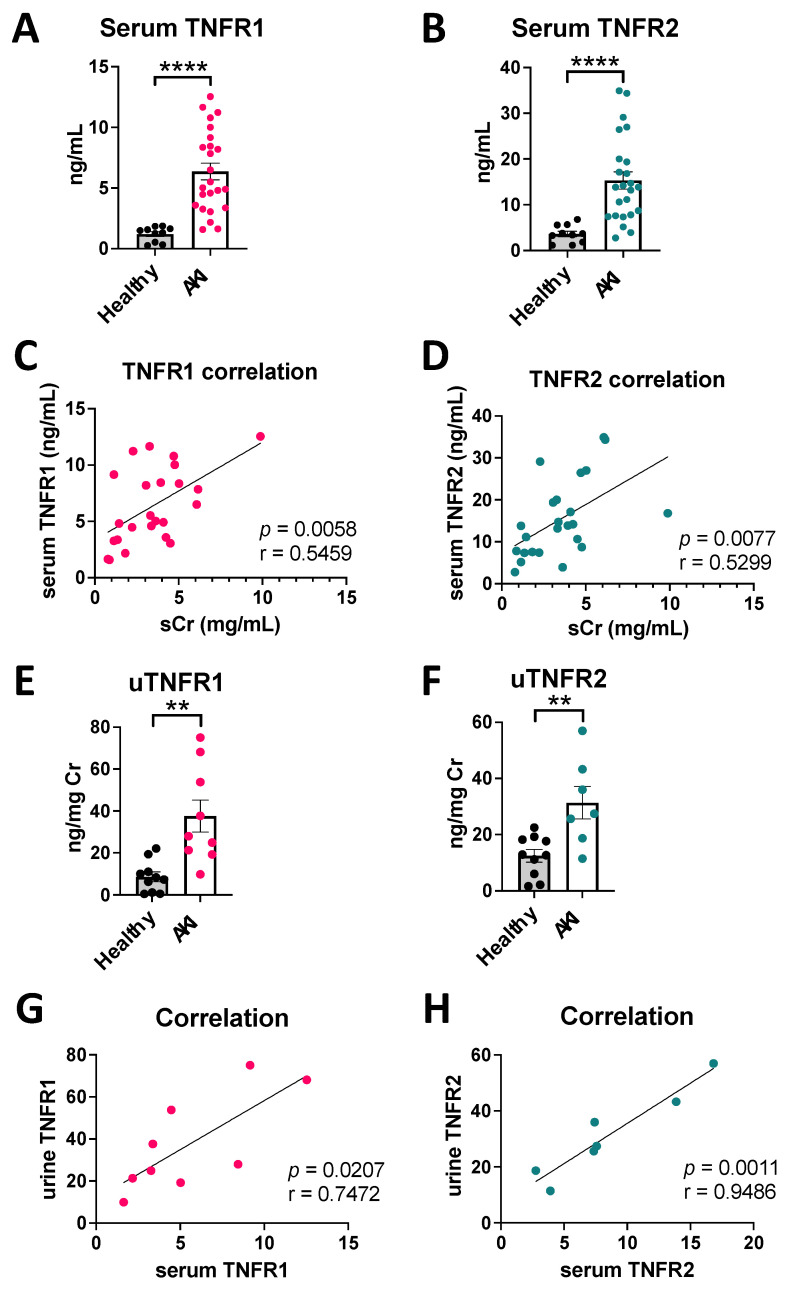
cTNFR1/2 are increased in human AKI and correlate with the severity of kidney failure. (**A**) Levels of cTNFR1 and (**B**) of cTNFR2 in healthy volunteer and AKI patient serum samples. (**C**) Correlation of cTNFR1 with serum creatinine in AKI patients. (**D**) Correlation of cTNFR2 with serum creatinine in AKI patients. (**E**) Urinary TNFR1 and (**F**) urinary TNFR2 in healthy volunteer and AKI patient samples. (**G**) Correlation of urinary TNFR1 with serum TNFR1 and (**H**) of urinary TNFR2 with serum TNFR2, in AKI patient samples. Pearson correlation (r) and respective *p* values are shown in graphs (**C**,**D**,**G**,**H**). **: *p* < 0.01, ****: *p* < 0.0001.

We next measured the levels of TNFR1/2 in AKI patient samples. AKI induced significant upregulation of cTNFR1/2 serum levels, on average 4–5-fold over levels in healthy volunteers (Figure 3A,B). These serum elevations correlate with loss of kidney function, assessed by serum creatinine (Figure 3C,D). Similar to the mouse models, urinary TNFR1/2 levels are also elevated in AKI patients (Figure 3E,F) and correlate with corresponding serum levels (Figure 3G,H; see also uTNFR1/2 levels without correction for uCr and respective correlations in Supplementary Figure S1). These data show that human AKI results in significant cTNFR1/2 elevations that correlate with AKI severity.

### 3.3. TNF Neutralization Does Not Affect Early cTNFR1/2 Serum Elevations after Kidney Injury

**Figure 4:**
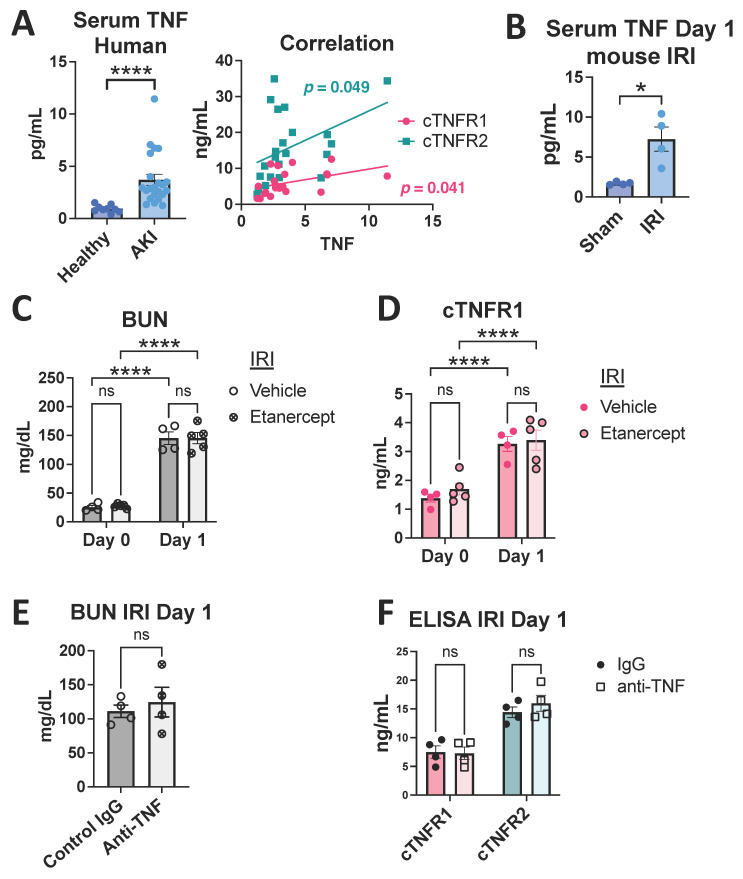
TNF neutralization does not affect cTNFR1/2 elevations early after bilateral IRI. (**A**) Serum TNF levels in healthy volunteers or AKI patients (left panel) and correlation with cTNFR1 and cTNFR2 in AKI (right panel). (**B**) Mouse serum TNF levels at day 1 post bilateral IRI or sham. (**C**) BUN levels at day 0 (before injury) and day 1 post bilateral IRI, in etanercept- or vehicle-treated mice. (**D**) cTNFR1 levels at day 0 (before injury) and day 1 post bilateral IRI in etanercept- or vehicle-treated mice. (**E**) BUN levels at day 1 post bilateral IRI in anti-TNF-antibody-or IgG-treated mice (**F**) cTNFR1 and cTNFR2 levels at day 1 post bilateral IRI in anti-TNF-antibody- or IgG-treated mice. *: *p* < 0.05, ****: *p* < 0.0001, ns: not significant.

It has been speculated, although not experimentally tested, that cTNFR1/2 are released as a feedback response to the activation of their cellular forms by their ligand, TNF, representing markers of TNF activity in kidney or other diseases [22,23]. Indeed, TNF circulating levels are increased acutely after kidney injury, as seen in our AKI patients and in the severe bilateral IRI mouse model (Figure 4A,B). To determine the contribution of TNF to cTNFR1/2 elevations in AKI, we neutralized TNF starting before the injury and examined cTNFR1/2 at day 1 post severe IRI. We found that mice pre-treated with murine etanercept [29] or an anti-TNF neutralizing antibody showed similar levels of kidney injury (Figure 4C,E) and similar cTNFR1/2 elevations (Figure 4D,F) compared to mice pre-treated with their respective controls (cTNFR2 levels were not evaluated after etanercept (TNFR2-Fc fusion molecule) treatment because of its interference with endogenous TNFR2 detection). We thus conclude that signaling induced by TNF does not mediate early cTNFR1/2 elevations post AKI.

### 3.4. Early cTNFR1/2 Elevations after AKI Can Be Attributed to Extrarenal Sources

**Figure 5:**
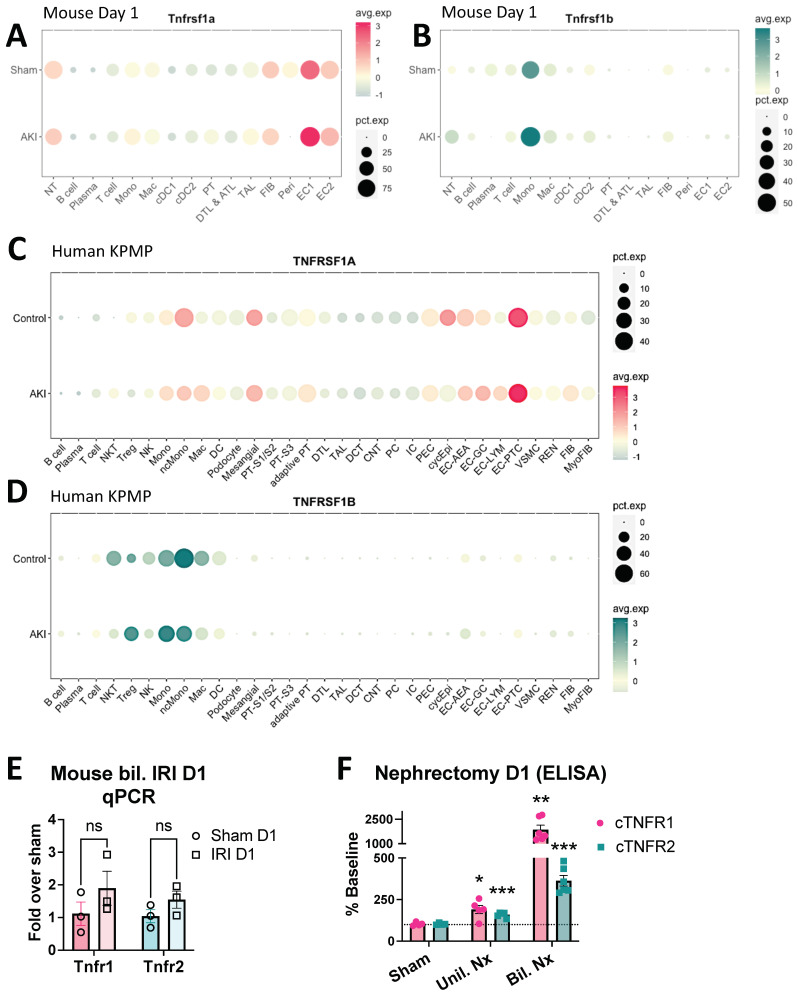
Kidney TNFR1/2 levels are slightly upregulated in AKI while nephrectomy significantly increases circulating TNFR1/2. (**A**) Dot plot of TNFR1 (Tnfrsf1a) expression in mouse kidney cell types from sham and AKI (bil. IRI day 1) samples. (**B**) Dot plot of TNFR2 (Tnfrsf1b) expression in mouse kidney cell types from control and AKI (bil. IRI day 1) samples. (**C**) Dot plot of TNFR1 (Tnfrsf1a) expression in human kidney cell types from control and AKI patient samples. (**D**) Dot plot of TNFR2 (Tnfrsf1b) expression in human kidney cell types from control and AKI patient samples. (**E**) qPCR analysis of TNFR1 and TNFR2 expression in whole kidney mRNA samples at day 1 post bilateral IRI or sham. (**F**) cTNFR1 and cTNFR2 levels at day 1 post sham surgery, unilateral nephrectomy (Unil. Nx), or bilateral nephrectomy (Bil. Nx), expressed as a percent of their respective baseline (dotted line denotes baseline). *: *p* < 0.05, **: *p* < 0.01, ***: *p* < 0.001, ns: not significant.

The injured kidney is a potential source of cTNFR1/2. To identify which kidney cell types express TNFR1 and TNFR2, we interrogated our existing single-cell RNAseq dataset of sham and IRI kidneys collected 24 h post injury [29,30]. At this time point, very strong elevations of serum and urine TNFR1/2 can be detected (Figure 1B,C and Figure 2A,B). We found that TNFR1 (*Tnfrsf1a*) is ubiquitously expressed in the kidneys of sham mice, with the highest levels detected in endothelial cells (EC1 and EC2), neutrophils (NT), fibroblasts (Fib), pericytes (Peri), monocytes (Mono), and macrophages (ΜΦ) (Figure 5A). Interestingly, 24 h after kidney injury we identified only a mild upregulation of TNFR1 expression in ECs and very mild upregulation of NT, monocytes, and some tubular cells (PT and TAL) (Figure 5A). In sham animals, TNFR2 (*Tnfrsf1b*) is mostly expressed in immune cells, mainly in monocytes (Figure 5B). Twenty-four hours after injury, TNFR2 expression increases slightly in monocytes, neutrophils (NT), and macrophages (ΜΦ) (Figure 5B). We next examined TNFR1/2 kidney expression profiles in human AKI using single-cell analysis datasets from the Kidney Precision Medicine Project (KPMP) atlas (https://www.kpmp.org/; accessed on 24 March 2022). The datasets include 20 healthy donor and 12 AKI patient samples. We found a ubiquitous expression of TNFR1 (*Tnfrsf1a*) in healthy donor samples and mild or no increases in AKI samples (Figure 5C). TNFR2 (*Tnfrsf1b*) expression was detected mainly in immune cells, and overall decreased (with mild increases in regulatory T cells (Treg) and some monocytes) in the AKI samples (Figure 5D). We finally tested total mouse kidney TNFR1/2 expression by qPCR. We found that neither receptor’s expression was significantly elevated in kidneys at day 1 post IRI (Figure 5E).

Considering the strong serum and urinary elevations we detected in mouse models and in human AKI (Figure 1 and Figure 3), the above results suggest that cTNFR1/2 are released from existing receptors’ pools that do not require transcriptional upregulation and/or that they are released from extrarenal sources. To test cTNFR1/2 elevations after loss of kidney function and simultaneously eliminate kidney contributions to cTNFR1/2 levels, we utilized unilateral or bilateral nephrectomies. We found that unilateral or bilateral nephrectomy results in significant cTNFR1/2 increases after 24 h (Figure 5F). These results show that extrarenal sources can contribute significant amounts of cTNFR1/2 during loss of kidney function.

### 3.5. cTNFR1/2 Levels and Their Kidney Expression Levels Remain Elevated for Weeks Post Kidney Injury

**Figure 6:**
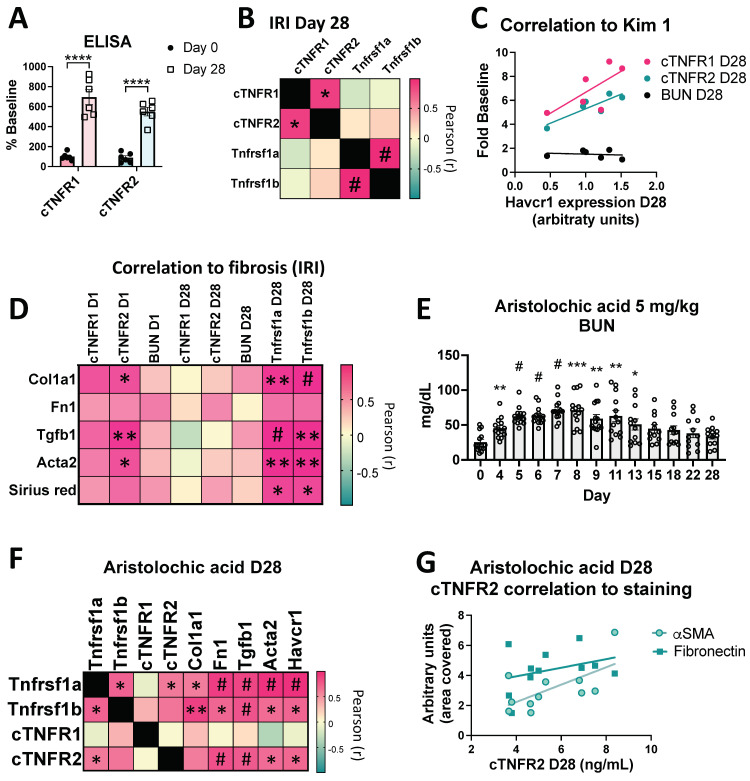
cTNFR1/2 and their kidney expression levels remain upregulated for weeks post injury and correlate with kidney injury and progression to fibrosis. (**A**) cTNFR1 and cTNFR2 levels, expressed as percent control (before injury), at 0 or 28 days post bilateral IRI (****: *p* < 0.0001) (**B**) Pearson r value heatmap of correlations between cTNFR1, cTNFR2, and their respective kidney mRNA expression levels, at 28 days post bilateral IRI (*: *p* < 0.05, #: *p* < 0.001). (**C**) Correlations between cTNFR1, cTNFR2, and BUN (expressed as fold over their baseline levels, *y*-axis) with kidney Kim1 (*Havcr1*) mRNA expression levels (*x*-axis). (**D**) Pearson r value heatmap of BUN, cTNFR1, and cTNFR2 at day 1 and day 28 post IRI and of TNFR1 and TNFR2 (*Tnfrsf1a* and *Tnfrsf1b* genes, respectively) kidney expression at day 28 post IRI with fibrosis marker kidney expression levels and Sirius red collagen staining (*: *p* < 0.05, **: *p* < 0.01, #: *p* < 0.001). (**E**) Time course of BUN levels in mice treated with a low dose (5 mg/kg) of aristolochic acid (*: *p* < 0.05, **: *p* < 0.01, ***: *p* < 0.001, #: *p* < 0.0001). (**F**) Pearson r value correlation heatmap of kidney expression levels of TNFR1 (*Tnfrsf1a*), TNFR2 (*Tnfrsf1b*), and of cTNFR1 and cTNFR2 with Kim1 and pro-fibrotic kidney gene expression, all at day 28 of the aristolochic acid model *: *p* < 0.05, **: *p* < 0.01, #: *p* < 0.001). (**G**) Correlations between cTNFR2 levels (*x*-axis) and kidney interstitial αSMA and fibronectin levels, quantified after immunostaining (*y*-axis).

Testing the long-term effects of kidney injury on cTNFR1/2 levels, we found that cTNFR1/2 remain elevated for at least four weeks post severe IRI (Figure 6A, C57Bl/6J mice; Supplementary Figure S2A, FVB/N mice), while traditional kidney function markers (such as BUN and serum creatinine) return to near baseline levels two to four weeks post injury (depending on the initial injury and the mouse strain, Supplementary Figure S2A) [25,29]. We have previously reported elevations in the kidney expression of TNFR1/2, beginning at day 2 post bilateral IRI, and at day 7 in the UUO model [25]. Analysis of a publicly available bulk RNA-seq [34] dataset confirmed a significant kidney TNFR2 expression increase at day 7 post bilateral IRI that remained for 12 months (Supplementary Figure S2B), while creatinine levels returned to normal at 14 days post injury in that study [34]. However, we found no significant correlation between cTNFR1/2 levels and kidney expression levels of each receptor (*Tnfrsf1a* and *Tnfrsf1b* genes, respectively) at day 28 post severe IRI (Figure 6B), suggesting again that kidneys do not represent the major source of cTNFR1/2.

### 3.6. Sustained cTNFR1/2 Elevations Correlate with Persistent Injury and Progression to Fibrosis in AKI-to-CKD Models

As a next step, we examined the hypothesis that cTNFR1/2 elevations correlate with AKI outcomes, in particular AKI-to-CKD progression and kidney fibrosis. Our severe IRI model leads to the development of fibrosis by three to four weeks post injury, assessed by increased interstitial collagen accumulation and fibrotic-marker gene upregulation, and loss of GFR, which becomes detectable 2–3 months post IRI [25,29,34,35]. Kidney injury molecule 1 (Kim1) is undetected in the healthy kidney and upregulated in injured proximal tubular cells after kidney injury and during transition to fibrosis. We found that cTNFR1/2 at day 28 post injury correlate with kidney Kim1 gene expression (Kim1 gene name: *Havcr1*) at the same time point, suggesting that sustained cTNFR1/2 levels indicate sustained tubular injury (Figure 6C; Pearson correlation r is 0.7030 and 0.8656 for cTNFR1 and cTNFR2, respectively, significant for cTNFR2: *p* = 0.026). Of note, the traditional kidney injury marker, BUN, shows no such correlation (Figure 6C); thus, cTNFR1/2 levels can be very informative in identifying remaining kidney injury. Moreover, we examined the relationship of cTNFR1/2 levels with fibrosis markers gene expression levels (collagen 1α: *Col1a1*; fibronectin: *Fn1*; TGFβ: *Tgfb1*; α smooth muscle actin: *Acta2*) and collagen deposition (assessed by Sirius red staining, see representative staining images in Supplementary Figure S2C), at day 28 post IRI (Figure 6D). cTNFR1/2 serum levels at day 28 do not correlate with those markers and neither do BUN levels (Figure 6D, columns 4–6). However, cTNFR1/2 serum levels at day 1 (in particular cTNFR2 levels) were predictive of subsequent fibrosis marker induction at day 28 (Figure 6D, columns 1–2). Again, BUN at day 1 failed to show any correlation with kidney fibrosis at day 28 (Figure 6D, column 3). Of note, kidney TNFR1/2 expression levels (*Tnfrsf1a* and *Tnfrsf1b*, respectively) are also increased at day 28 post injury and very strongly correlate with all fibrosis markers (Figure 6D, columns 7–8), suggesting that the mechanisms that drive TNFR1/2 kidney expression and development of fibrosis are related.

In a different AKI-to-CKD model, low dose (5 mg/kg) aristolochic acid (AA) shows gradual elevations of BUN, which peak at Day 7 post AA injection (Figure 6E), and kidney fibrosis development at day 28 [24,35], a time point at which BUN has returned to baseline. Peak BUN (day 7) or day 28 BUN levels do not correlate with fibrosis gene expression at the endpoint, day 28 (Supplementary Figure S2D,E). Interestingly, we found a significant correlation of cTNFR2 (but not of cTNFR1) serum levels at day 28 with the tubular injury marker Kim1 (*Havcr1*), with kidney fibrosis markers fibronectin (*Fn1*), TGFβ, and α, and smooth muscle actin (*Acta2*), as well as a positive, although not significant, correlation with collagen 1α (*Col1a1*) expression (Figure 6F). Again, we see significant correlations of TNFR1 (*Tnfrsf1a*) and TNFR2 (*Tnfrsf1b*) kidney mRNA expression at day 28, with all fibrosis marker levels tested and with Kim1 expression (Figure 6F). We finally examined the kidney interstitial levels of the profibrotic markers fibronectin and alpha smooth muscle actin (αSMA) with immunostaining, and found a positive correlation with cTNFR2 levels (Figure 6G; Pearson correlation r is 0.6412 and 0.3352 for αSMA and fibronectin, respectively, significant for αSMA: *p* = 0.0335), but not with cTNFR1 (not shown) in this model. Of note, there was again no correlation between kidney expression levels of TNFR1 and cTNFR1 and between kidney expression levels of TNFR2 and cTNFR2, suggesting that extrarenal tissues are the main circulating receptors’ sources at day 28 in the AA model (Figure 6F).

Taken together, these results suggest that kidney-injury-induced sustained TNFR1/2 serum elevations indicate remaining kidney injury and that TNFR1/2 kidney expression and/or their circulating serum levels, particularly cTNFR2 levels, correlate with progression to kidney fibrosis.

## 4. Discussion

In this study, we experimentally tested the effect of kidney injury on cTNFR1/2 levels. We found that kidney injury causes acute cTNFR1/2 increases, beginning within one hour post injury, with maximal levels around 24 h post IRI. These elevations correlate with kidney function loss and are detected for prolonged periods, measured up to one month in our AKI-to-CKD mouse models. These results align with a recent report showing that CKD patients from the Chronic Renal Insufficiency Cohort (CRIC) Study that had an AKI episode showed increased cTNFR1/2 levels compared to CKD patients that did not have an AKI episode, and these elevations were detected months after the AKI episode [16]. It is intriguing that sustained cTNFR2, and to a lower degree cTNFR1, levels correlate with kidney Kim1 expression, an established and specific marker of tubular injury. Thus cTNFR1/2 elevations, in contrast to traditional markers of kidney function, such as BUN, are markers of persistent kidney injury during AKI-to-CKD transition. In addition, cTNFR2 levels (and to a lesser degree cTNFR1 levels) early after injury (day 1) correlate with kidney fibrosis markers levels during the transition to CKD (day 28). This is reminiscent of the predictive properties of cTNFR1/2 serum levels in CKD progression reported in numerous human studies. Based on the strong correlation of cTNFR1/2 levels with kidney function loss in AKI, their significant elevations in unilateral kidney injury models, their sustained upregulation in AKI-to-CKD progression, and their correlation with injury and fibrosis markers, we conclude that cTNFR1/2 serum levels represent sensitive markers of kidney function loss and underlying injury, outperforming BUN and serum creatinine. Whether this property is what renders them predictive markers of kidney disease progression in CKD or whether they also have disease-modulating functions in AKI or CKD is not yet clear.

TNFR1/2 kidney expression levels change only slightly 24 h after injury in mice or in AKI patients, suggesting release by extrarenal sources. Although kidney TNFR1/2 expression is increased at later time points, receptors’ kidney expression does not correlate with their respective serum levels in any of our models, suggesting again extrarenal contributions. This is also corroborated by the fact that bilateral nephrectomies lead to very strong serum elevations of cTNFR1/2. These findings agree with a diabetic nephropathy study showing a lack of correlation between circulating and kidney expression levels of TNFR1/2, using biopsies of patients obtained within six months before/after their cTNFR1/2 measurements [9]. Moreover, TNF neutralization does not affect early AKI cTNFR1/2 elevations, consistent with CKD studies that show correlation of cTNFR1/2 but not TNF (total or free) with CKD progression [1,2], suggesting different regulation mechanisms of the ligand’s versus the receptors’ serum levels. The exact cTNFR1/2 sources and release mechanisms require further investigation.

A potential explanation for the observed cTNFR1/2 serum elevations is the compromised circulating receptor clearance in kidney injury or after nephrectomy. This explanation is not likely the sole mechanism of cTNFR1/2 increases because (a) we detect significant elevations of TNFR1/2 in the urine in AKI, suggesting an increase in the total amount of receptors; (b) we detect increased cTNFR1/2 in models of unilateral injury, in which the remaining healthy kidney compensates for functional output, hence no increase in functional biomarkers (e.g., BUN, creatinine) is detected; and, (c) the uremic toxin indoxyl sulfate causes acute cTNFR1/2 elevations in healthy mice (normal kidney function), suggesting the activation of cTNFR1/2 release mechanisms by uremia. This conclusion is further supported by CKD patients’ studies, which show increased levels of cTNFR1/2 and predictive value for disease progression even after correcting for eGFR, suggesting that their elevations are not merely a result of decreased clearance [1,2].

Based on their significant predictive value in CKD progression and the fact that their elevations are detected long before the development or progression of CKD, cTNFR1/2 could potentially have a causal role in disease progression. However, mechanistic studies exploring these functions in the context of in AKI, CKD, or AKI-to-CKD transition have not yet been reported. An important impediment is the lack of knowledge of the molecular mechanisms of cTNFR1/2 release in kidney disease. Our work provides novel insights into kidney-injury-induced cTNFR1/2 elevations that may enable future mechanistic studies aiming for a better understanding of cTNFR1/2 biology and potential roles in kidney disease progression.

## Data Availability

Human single-cell sequencing data were generated by KPMP: DK133081, DK133091, DK133092, DK133093, DK133095, DK1330971, DK114866, DK114908, DK133090, DK133113, DK133766, DK133768, DK114907, DK114920, DK114923, DK114933, DK114886; https://www.kpmp.org, accessed on 24 March 2022. All other data underlying this article are available in the article and in its online supplementary material.

## Supplementary Materials

The following supporting information can be downloaded at: https://www.mdpi.com/article/10.3390/cells12182214/s1, Figure S1: Urinary TNFR1 and TNFR2 concentration in AKI patients without urinary creatinine correction and their correlation to cTNFR1 and cTNFR2, respectively; Figure S2: Long-term TNFR1/2 elevations after injury and BUN correlations to fibrosis markers; Table S1: qPCR primer sequences.
