# Supplementary material for: Acute Kidney Injury-Induced Circulating TNFR1/2 Elevations Correlate with Persistent Kidney Injury and Progression to Fibrosis
Source: Cells. 2023 Sep 5;12(18):2214. doi: 10.3390/cells12182214 (PMC10527245; doi:10.3390/cells12182214)
Supplement: Supplementary file 1 [file cells-12-02214-s001.zip › cells-2570149-supplementary.pdf]

Supplemental Files

Table of contents:

1. **Supplemental Figure S1:** Urinary TNFR1 and TNFR2 concentration in AKI patients without urinary creatinine correction and their correlation to cTNFR1 and cTNFR2 respectively.
2. **Supplemental Figure S2:** Long-term TNFR1/2 elevations after injury and BUN correlations to fibrosis markers
3. **Supplemental Table S1.** qPCR primer sequences

Supplemental Figure S1: A. Urinary TNFR1 and TNFR2 concentration in AKI patients without urinary creatinine correction and, B. uTNFR1 and uTNFR2 correlation to cTNFR1 and cTNFR2 respectively.

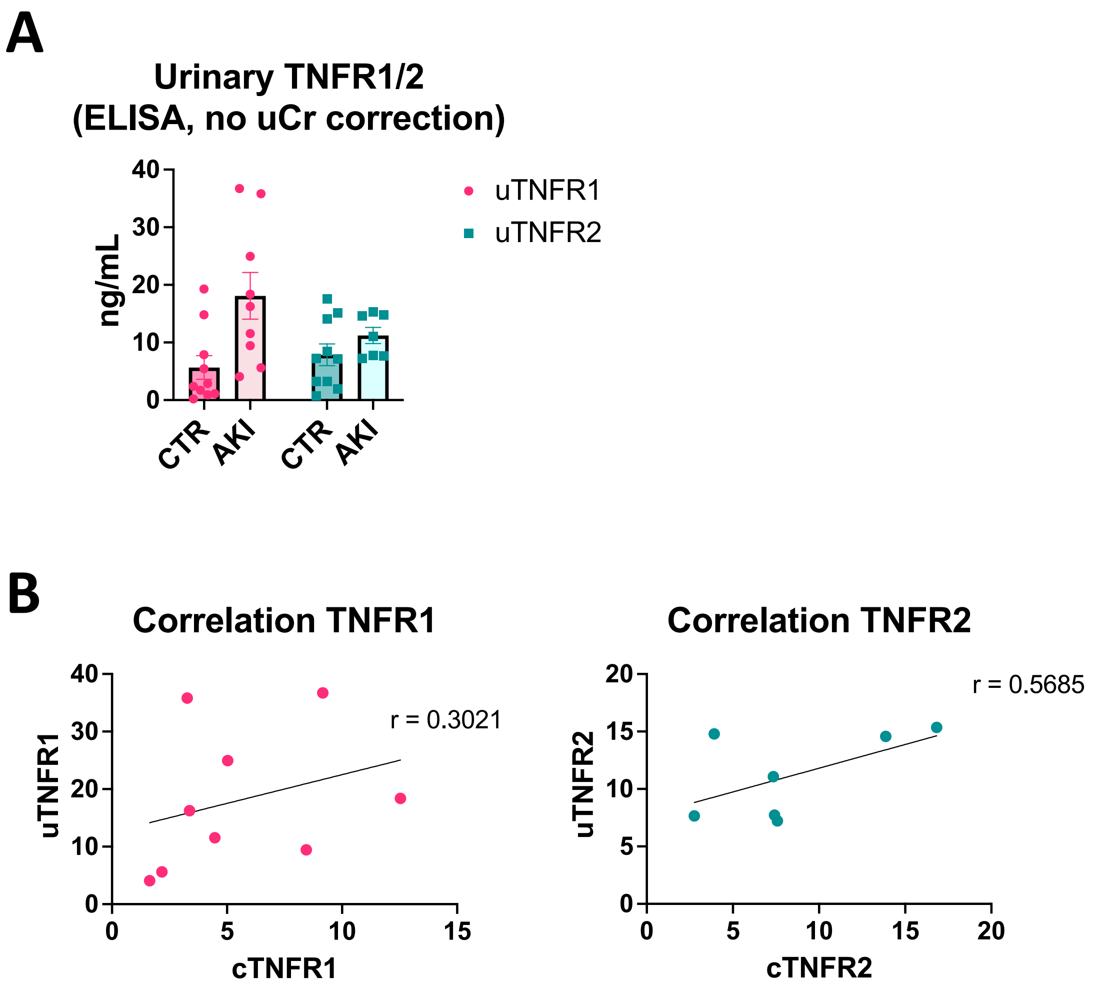

Supplemental Figure S2: Long-term TNFR1/2 elevations after injury and BUN correlation to fibrosis markers. A. Sustained cTNFR1/2 elevations at day 28 post injury in the FVB/N mouse strain. B. Bulk mRNA sequencing analysis showing sustained kidney TNFR2 mRNA expression after IRI. C. Representative images of low and high levels of Sirius red staining at day 28 post IRI. D. Peak BUN (day 7) levels do not correlate to fibrosis/injury gene expression (day 28) in the aristolochic acid injury model E. BUN day 28 levels do not correlate to fibrosis/injury gene expression at day 28 of the aristolochic acid injury model.

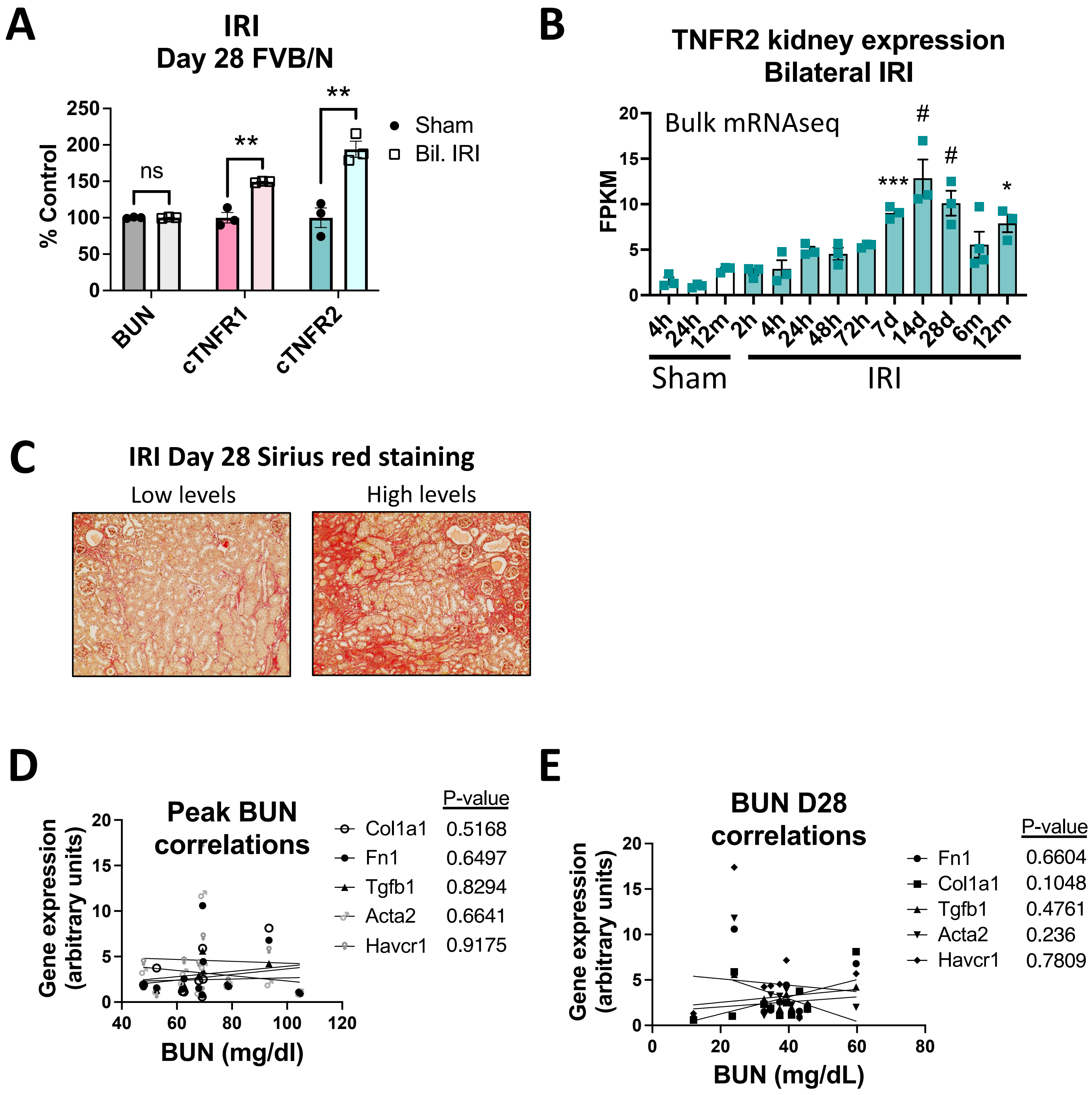

Supplemental Table S1. qPCR primer sequences

Target gene	Synonym	Direction	Sequence
**Acta2**	αSMA	Forward	AGCCATCTTTCATTGGGATGGA
**Acta2**	αSMA	Reverse	TACCCCCTGACAGGACGTTG
**Col1a1**	collagen 1a	Forward	CCTGACGCATGGCCAAGAAG
**Col1a1**	collagen 1a	Reverse	TACCTCGGGTTTCCACGTCT
**Fn1**	fibronectin	Forward	GGTTCGGGAAGAGGTTGTGA
**Fn1**	fibronectin	Reverse	CACTCCTCTCCAATGGCGTA
**Gapdh**		Forward	ACCACAGTCCATGCCATCAC
**Gapdh**		Reverse	TCCACCACCCTGTTGCTGTA
**Havcr1**	Kim1	Forward	ACAACAGCTGAGGTGACAGG
**Havcr1**	Kim1	Reverse	TTCCCTGGAGGGATTGCTTC
**Tgfb1**	TGFβ	Forward	CTGCTGACCCCCACTGATAC
**Tgfb1**	TGFβ	Reverse	AGCCCTGTATTCCGTCTCCT
**Tnfrsf1a**	TNFR1	Forward	ACGAATCACTCTGCTCCGTG
**Tnfrsf1a**	TNFR1	Reverse	TCGCAAGGTCTGCATTGTCA
**Tnfrsf1b**	TNFR2	Forward	GAGATGCCAAGGTGCCTCAT
**Tnfrsf1b**	TNFR2	Reverse	AACTGGGTGCTGTGGTCAAC
